# Anti-Tumor Activities of Anti-Siglec-15 Chimeric Heavy-Chain Antibodies

**DOI:** 10.3390/ijms26115068

**Published:** 2025-05-24

**Authors:** Kexuan Cheng, Jiazheng Guo, Yating Li, Qinglin Kang, Rong Wang, Longlong Luo, Wei Wang, Jiansheng Lu

**Affiliations:** 1 Laboratory of Advanced Biotechnology, Beijing Institute of Biotechnology, Beijing 100081, China; 16696653458@163.com (K.C.); sdqzgjz@163.com (J.G.); lyt18214729367@163.com (Y.L.); kql_lynn@163.com (Q.K.); wangrong_8312@163.com (R.W.); 2 College of Public Health, Zhengzhou University, Zhengzhou 450001, China; 3 State Key Laboratory of Toxicology and Medical Countermeasures, Institute of Pharmacology and Toxicology, 27 Taiping Road, Beijing 100850, China; luolong_long@126.com

**Keywords:** Siglec-15, nanobody, phage display technology, checkpoint blockade immunotherapy, chimeric heavy-chain antibody

## Abstract

Immune checkpoint inhibitors like programmed cell death 1 (PD-1) antibodies have revolutionized cancer treatment, but patient response rates remain limited. Sialic acid-binding Ig-like lectin 15 (Siglec-15) has emerged as a promising new immune checkpoint target. Through phage display technology using a Bactrian camel immunized with recombinant human Siglec-15, we generated six anti-Siglec-15 camelid nanobodies and constructed chimeric heavy-chain antibodies by fusing the VHH domains with human IgG-Fc. Following expression in HEK293-F cells and purification, three antibodies (S1, S5, S6) demonstrated specific binding to both human and murine Siglec-15 in ELISA and biolayer interferometry assays. In a xenograft model established by subcutaneous inoculation of NCI-H157-S15 cells into BALB/c nude mice, these antibodies showed distinct tumor targeting and significant blockade of Siglec-15 interactions with CD44, MAG, sialyl-Tn, and LRR4C ligands. All three antibodies exhibited anti-tumor effects, with S1 showing the most potent activity. S1-treated mice had significantly smaller tumor volumes and weights compared to controls. The S1, S5, and S6 treatment groups showed enhanced anti-tumor immunity, with reduced TGF-β, IL-6, and IL-10 levels. Notably, S1 treatment significantly increased tumor-associated macrophages in tumor tissues (*p* < 0.05). In conclusion, S1 exhibits remarkable anti-tumor activity and has the potential to be developed as a cancer immunotherapy targeting Siglec-15.

## 1. Introduction

The evolution of cancer therapy has witnessed a paradigm shift with the emergence of tumor immunotherapy as the fourth pillar of cancer treatment, alongside surgery, radiotherapy, and chemotherapy [1], offering new hope for patients with advanced-stage tumors. Immunotherapy, targeting the immune checkpoints Programmed cell death 1 (PD-1) and its ligand PD-L1, has been shown to prolong overall survival in patients with lung cancer [2]. Despite the promising efficacy and safety of antibodies targeting PD-1 and PD-L1 in treating tumors such as non-small cell lung cancer, melanoma, and ovarian cancer, current tumor immunotherapy approaches still face numerous challenges, including unresolved issues related to primary and acquired resistance [3].

In this context, sialic acid-binding Ig-like lectin 15 (Siglec-15) has emerged as a novel immunosuppressive factor through genome-scale T cell activity screening and pan-cancer bioinformatic analyses [4]. Structurally classified as a type I transmembrane protein within the immunoglobulin superfamily, Siglec-15 exhibits high evolutionary coservation and a unique functional profile [5]. Unlike PD-L1, which primarily mediates T cell exhaustion through apoptosis-inducing pathways, Siglec-15 primarily promotes tumor immune escape by inhibiting T cell function via mechanisms centered on cellular growth regulation. Siglec-15 is widely presents across diverse malignancies, including lung cancer, colon cancer, and liver cancer, while remaining nearly undetectable in most healthy tissues [6,7]. This tumor-restricted expression pattern positions Siglec-15 as an attractive therapeutic target with potential for reduced on-target, off-tumor toxicity. Critically, Siglec-15 and PD-L1 demonstrate mutually exclusive expression profiles in the tumor microenvironment, suggesting that Siglec-15-targeted therapies could address a critical unmet need in patients refractory to PD-1/PD-L1 inhibition [8,9]. These features, combined with its homology to PD-L1 in structural domains and immunosuppressive function, have propelled Siglec-15 to the forefront of next-generation immune checkpoint research.

In this study, we isolated and characterized six novel anti-Siglec-15 chimeric heavy-chain antibodies derived from a phage-display camelid nanobody library. Through a series of in vitro and in vivo validations, we demonstrate their capacity to specifically bind Siglec-15, disrupt its immunosuppressive signaling, and elicit potent anti-tumor responses. These findings provide a foundation for translating this therapeutic strategy into clinical development. By targeting a pathway distinct from PD-1/PD-L1, these antibodies offer a potential new treatment option for cancer patients, particularly those resistant to existing checkpoint inhibitors. Future efforts will focus on optimizing these candidates for clinical application, evaluating combinatorial approaches with established therapies, and elucidating biomarkers to guide patient stratification.

## 2. Results

### 2.1. Construction and Screening of the Phage Display Nanobody Library

**Figure 1:**
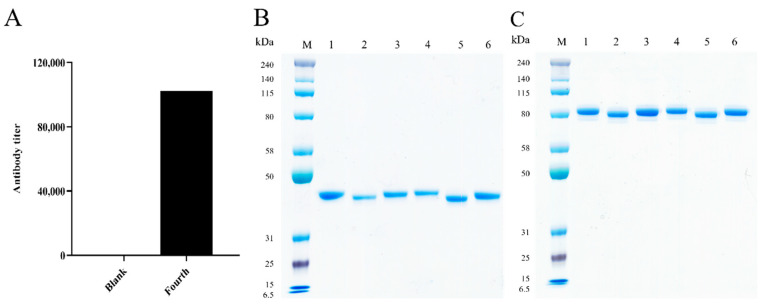
(**A**) Detection of the antibody titer in camel serum. The specific titer of the antibody against Siglec-15 in camel serum was detected before immunization and after the fourth immunization. (**B**) SDS-PAGE analysis of six purified chimeric heavy chain antibodies. Analysis of the six chimeric heavy chain antibodies by SDS-PAGE under reducing conditions. (**C**) Analysis of the six chimeric heavy chain antibodies by SDS-PAGE under non-reducing conditions. M: protein molecular weight standard, 1~6: purified S1~S6.

After immunization with Siglec-15, the camel serum exhibited a significant increase in Siglec-15-specific antibodies, reaching a titer of 1: 102,400 (Figure 1A). Subsequently, we constructed a phage display nanobody library, with a capacity of 4.1 × 10^9^. Three rounds of screening under progressively more stringent conditions effectively enriched clones that specifically bound to Siglec-15 (Table 1). In total, 370 positive clones were identified from 423 clones using phage-ELISA. After sequencing and analysis, six sequences of Siglec-15-specific nanobodies were obtained.

### 2.2. Generation of the Chimeric Heavy-Chain Antibodies

The obtained gene fragments were fused with the human IgG1 Fc (hFc) sequence. After expression and purification, six chimeric heavy-chain antibodies were obtained, designated as S1, S2, S3, S4, S5, and S6. SDS-PAGE analysis revealed that the molecular weight of these heavy-chain antibodies was 40 kDa under reducing conditions (Figure 1B) and 80 kDa under non-reducing conditions (Figure 1C), which matched the theoretical molecular weight of the heavy-chain antibody.

### 2.3. Characterization of the Chimeric Heavy-Chain Antibodies

**Figure 2:**
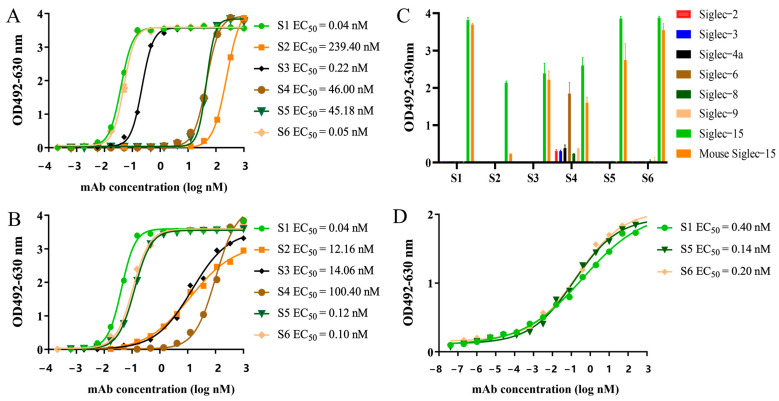
Specificity and binding activities of the chimeric heavy-chain antibodies. (**A**) Binding activities of S1-S6 to recombinant murine Siglec-15 protein. (**B**) Binding activities of S1–S6 to recombinant human Siglec-15 protein. (**C**) Specificity of S1–S6. (**D**) Binding activities of S1, S5, and S6 to NCI-H157-S15 cells. The experiments were repeated three times under this experimental condition.

Human-mouse cross-conjugated antibodies are more conducive to in vivo activity evaluation using mouse models. In the present study, we examined the binding activity of purified obtained antibodies to recombinant human and murine Siglec-15 proteins by ELISA. Antibodies bound to recombinant human and murine Siglec-15 proteins in a concentration-dependent manner, with S1, S3, and S6 having good binding activity to recombinant murine Siglec-15 protein, with 50% effect concentration (EC_50_) values ranging from 0.04 to 0.22 nM, S4 and S5 having poor binding activity to recombinant murine Siglec-15 protein, with EC_50_ values of around 45 nM, and S2 binding to the recombinant murine Siglec-15 protein had the worst binding ability (Figure 2A); S1, S5, and S6 had good binding activity with recombinant human Siglec-15 protein, with EC_50_ values ranging from 0.04 to 0.12 nM, and S4 had the worst binding activity with an EC_50_ value of 100.40 nM (Figure 2B). Notably, S1 and S6 demonstrated strong species cross-binding activity.

Antibody specificity is a crucial parameter in drug evaluation. Other proteins from the Siglecs family were selected as controls. As shown in Figure 2C, S1, S5 and S6 specifically bound to recombinant human and murine Siglec-15 proteins but not to control proteins. S2 bind to recombinant human Siglec-15 protein and weakly to recombinant murine Siglec-15 protein. S4 not only bound to recombinant human Siglec-15 protein, but also interacted with control proteins. Based on the binding activity and specificity assay results, we selected S1, S5, and S6 for subsequent evaluation. The binding of the antibody to Siglec-15 on the membrane surface of NCI-H157-S15+ cells was further detected by cellular ELISA, and the antibody binds to NCI-H157-S15+ cells in a concentration-dependent manner, suggesting that S1, S5, and S6 can efficiently bind to Siglec-15 expressed on the surface of the cells, with EC_50_ values ranging from 0.14 to 0.40 nM (Figure 2D).

**Figure 3:**
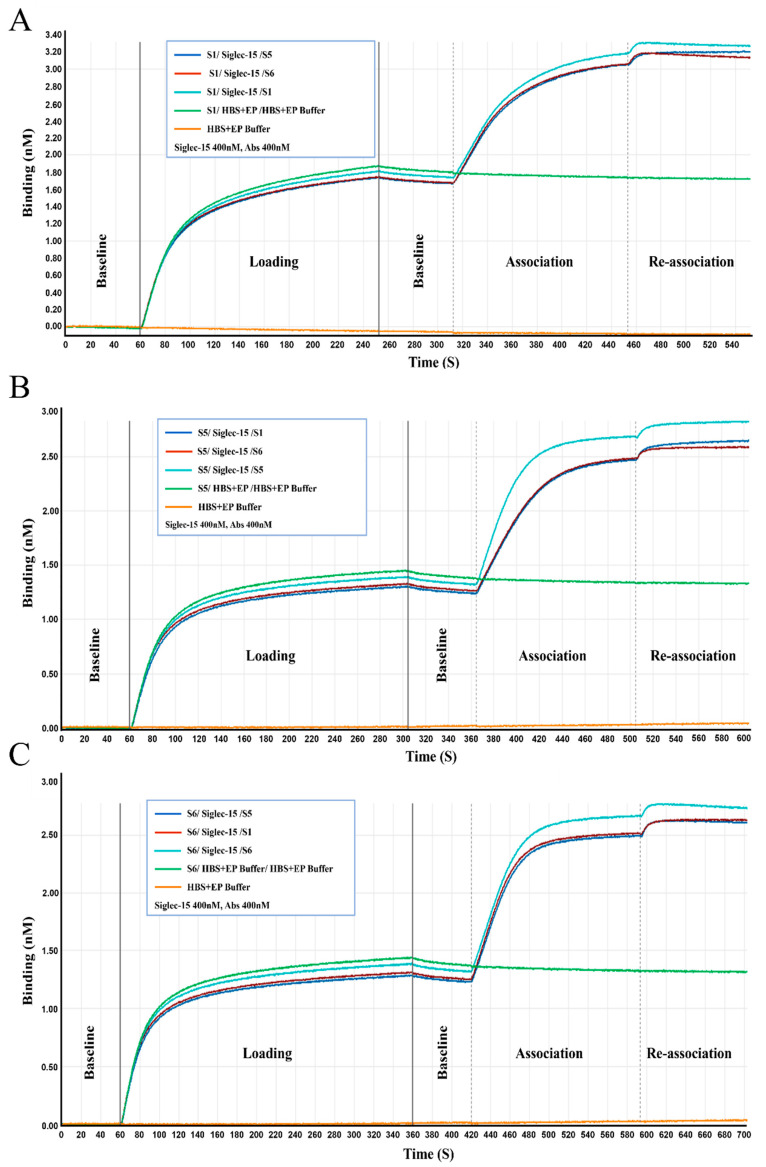
The kinetic process of competitive binding between S1 (**A**), S5 (**B**), and S6 (**C**) to recombinant human Siglec-15 protein. In boxed legends, samples are separated using “/” to represent the loading, association, and re-association stages of the process. The experiments were repeated three times under this experimental condition.

Additionally, the affinity and competitive binding of S1, S5, and S6 to recombinant human Siglec-15 protein were measured using BLI. The equilibrium dissociation constant (KD) values for the binding of S1, S5, and S6 to Siglec-15 were 0.13 nM, 0.16 nM, and 0.21 nM, respectively (Table 2 and Figure S1). The competition experiments demonstrated that during the re-association process, S1, S5, and S6 showed a minimal increase in amplitude and displayed consistent signal fluctuations. These observations indicated a competitive binding between S1, S5, and S6 and Siglec-15 (Figure 3).

All data were calculated using a 1:1 binding model in Analysis Software 7.0. Kon, association constant; Kdis, dissociation constant; KD, equilibrium dissociation constant; KD, Kdis/Kon.

### 2.4. Chimeric Heavy-Chain Antibodies’ Blocking Activity

**Figure 4:**
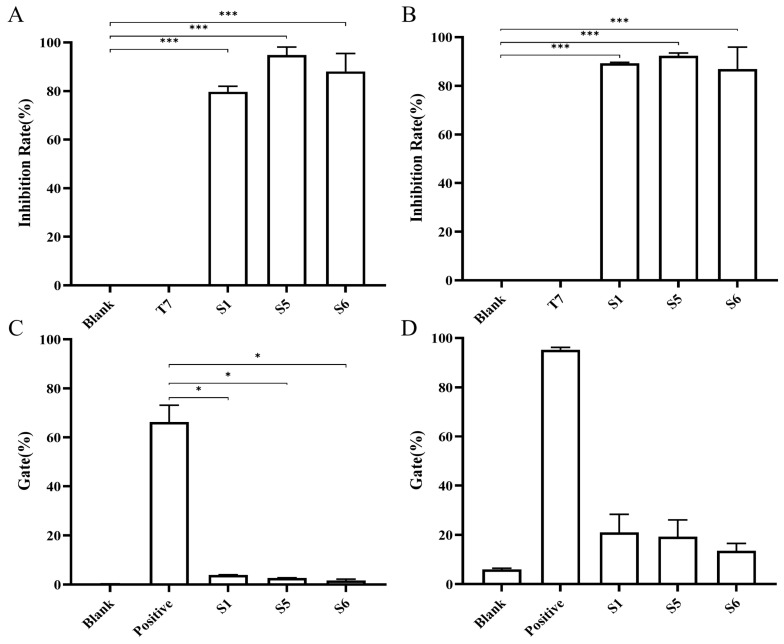
Blocking activities between the chimeric heavy-chain antibodies and different ligands of Siglec-15. The blocking rates of S1, S5, and S6 of the interaction between Siglec-15 and CD44 (**A**) or Siglec-15 and MAG (**B**). The positive group comprised biotin-labeled Siglec-15-Fc and HRP-labeled streptavidin, the blank group only contained HRP-labeled streptavidin, and T7 was used as the irrelevant antibody. The gate percentage of S1, S5, and S6 blocked the binding of sialyl-Tn (**C**), LRRC4C (**D**) and to Siglec-15. The positive group comprised biotin-labeled Siglec-15-Fc and APC-labeled streptavidin whereas the blank group only contained APC-labeled streptavidin. Error bar represents the standard error of the mean. The experiments were repeated two times under this experimental condition. The normality of data was assessed with Shapiro–Wilk test, and homogeneity of variance was evaluated using Levene’s test. If both assumptions were satisfied, one-way ANOVA followed by Tukey’s multiple comparison test was performed. For non-normally distributed or heteroscedastic data, the Kruskal–Wallis test was used, followed by Dunn’s multiple comparison test to assess intergroup differences. * *p* < 0.05, *** *p* < 0.001.

According to literature reports, the interaction of Siglec-15 with its ligands CD44, MAG, and sialyl-Tn mediates immune escape in the tumor microenvironment, thus affecting the development of tumors [10,11,12,13,14,15]. A previous study suggested that Siglec-15 binds to CD44, MAG, and LRRC4C independently of sialyl-Tn [16]. The results showed that S1, S5, and S6 all blocked the binding activity between Siglec-15 and CD44 at concentrations of 10 μg/mL and above, with blocking activities (inhibition rates) of 94.86% for S5, 88.05% for S6, and 79.69% for S1 (Figure 4A). S1, S5, and S6 could also block Siglec-15 from binding to MAG at concentrations of 10 μg/mL and above, with blocking activities of 92.37% for S5, 89.25% for S1, and 86.94% for S6 (Figure 4B). Flow cytometry was also used to assess whether S1, S5, and S6 could bock Siglec-15 from binding to sialyl-Tn. NCI-H157 cells, which express high surface levels of sialyl-Tn, were verified by employing an anti-sialyl-Tn fluorescent antibody (Figure S2). S1, S5, and S6 were able to effectively block the binding of Siglec-15 to sialyl-Tn (Figure 4C) and LRRC4C (Figure 4D).

### 2.5. Evaluation of Chimeric Heavy Chain Antibodies Mediating T Cell Proliferation and IFN-γ Secretion

**Figure 5:**
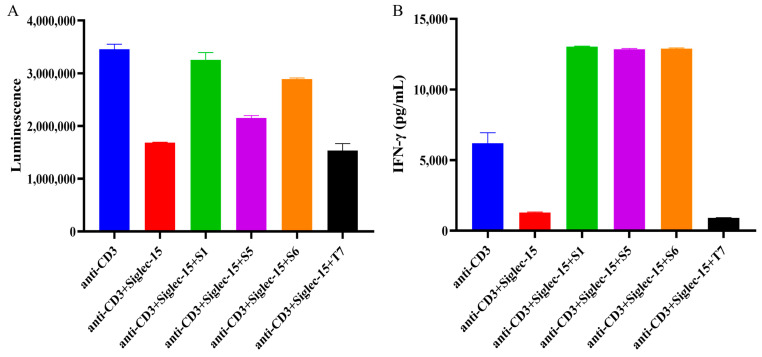
Biological activities of chimeric heavy-chain antibodies in vitro. (**A**) S1, S5, and S6 reversed the inhibition of PBMC lymphocyte proliferation by Siglec15 protein. (**B**) S1, S5, and S6 reversed the inhibition of PBMC lymphocyte IFN-γ secretion by Siglec15 protein. T7 was used as the irrelevant antibody. Error bar represents the standard error of the mean. The experiments were repeated two times under this experimental condition.

According to literature reports, CD3 monoclonal antibody specifically recognizes CD3 molecules on the surface of T cells, leading to T cell activation and proliferation through the binding of TCR-CD3 complexes on the surface of T cells to MHC-II class II molecules-antigenic peptides on the surface of antigen-presenting cells [17,18]. Siglec-15 binds to an unknown ligand on the surface of T cells and activates an inhibitory signaling pathway that suppresses T cell activation, leading to decreased proliferation and cytokine secretion (e.g., IFN-γ), and may also exacerbate T cell depletion [10,15]. In this study, we examined the reversal of Siglec-15-mediated inhibition of proliferation and secretion of IFN-γ by chimeric heavy chain antibodies in T cells. As shown in Figure 5, S1, S5, and S6 all reversed the Siglec-15-mediated inhibition of human T cells (Figure 5A) as well as the inhibition of IFN-γ secretion (Figure 5B) by T cells compared to the control antibody T7.

### 2.6. Evaluation of the Anti-Tumor Activity Mediated by the Chimeric Heavy-Chain Antibodies In Vivo

**Figure 6:**
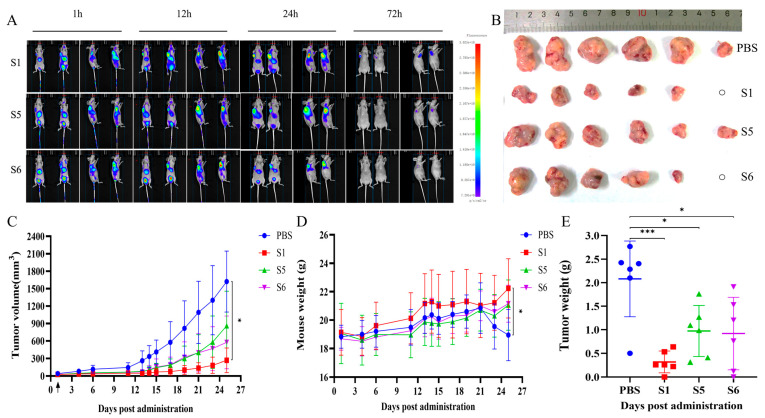
Biological activities of the chimeric heavy-chain antibodies in vivo. (**A**) Fluorescence distribution in vivo after tail vein injection of fluorescently labelled antibodies in xenograft model mice. (**B**) Images of tumors excised from each group. (**C**) Growth curve of tumor volume over time. Arrows indicated the first antibody injection (10 mg/kg, administered intravenously every 2 days). (**D**) Changes in mouse body weight. (**E**) Weights of tumors excised at the end of the treatment. The control group represented NCI-H157-S15 tumor xenograft mice treated with PBS. The S1, S5, and S6 groups represented tumor xenograft mice injected with antibodies intravenously. The normality of data was assessed with the Shapiro–Wilk test, and homogeneity of variance was evaluated using Levene’s test. If both assumptions were satisfied, one-way ANOVA followed by Tukey’s multiple comparison test was performed. For non-normally distributed or heteroscedastic data, the Kruskal–Wallis test was used, followed by Dunn’s multiple comparison test to assess intergroup differences. n = 6, * *p* < 0.05, *** *p* < 0.001.

BALB/c nude mice were subjected to subcutaneous inoculation of NCI-H157-S15 cells to construct a xenograft model, and then after tail vein injection of antibodies (labelled with fluorescence), mice were photographed using the Small Animal Live Imaging System at different time points. At 1 h after the administration of the antibodies, the antibodies were widely distributed in the areas of liver, bladder, and tumor tissues, and the fluorescence signals at the tumor sites were more significant. At 72 h after administration, fluorescent signals could still be observed at the tumor site in mice in lateral recumbency. This indicates that S1, S5, and S6 are able to target the tumor site in mice (Figure 6A). The anti-tumor activities of S1, S5, and S6 were evaluated in a xenograft model. Tumors in the antibody-treated groups were significantly smaller than those in the control group (Figure 6B). In comparison with the control (PBS) group, all treatment groups (S1, S5, and S6) showed anti-tumor activities, with the S1-treated group demonstrating a significant inhibitory activity on tumor growth (Figure 6C, *p* < 0.05). Mice in the control group exhibited a significant decrease in body weight compared with those in the S1-treated group (Figure 6D, *p* < 0.05). Tumor weight in the antibody-treated group was significantly less than in the control group. The average weights of the excised tumors at the end of the experiment were 0.3185 g in the S1 group, 0.9734 g in the S5 group, 0.9208 g in the S6 group, and 2.0795 g in the control group (Figure 6E). These findings suggested that S1, S5, and S6 all possessed anti-tumor activities in the xenograft mice, with S1 demonstrating the strongest activity.

**Figure 7:**
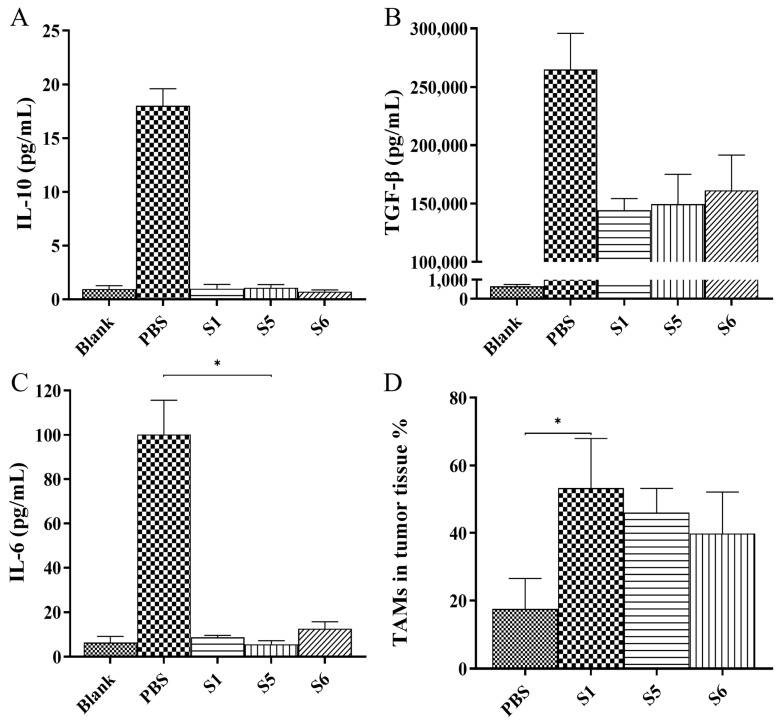
The level of cytokines in the peripheral blood serum of mice. (**A**) TGF-β. (**B**) IL-6. (**C**) IL-10. (**D**) The percentage of TAMs in tumor tissues. The blank group comprised BALB/c nude healthy female mice that were not inoculated with tumor cells. The normality of data was assessed with the Shapiro–Wilk test, and homogeneity of variance was evaluated using Levene’s test. If both assumptions were satisfied, one-way ANOVA followed by Tukey’s multiple comparison test was performed. For non-normally distributed or heteroscedastic data, the Kruskal–Wallis test was used, followed by Dunn’s multiple comparison test to assess intergroup differences. Error bar represents the standard error of the mean. n = 3, * *p* < 0.05.

In the tumor microenvironment, cytokines serve as essential molecules that facilitate signal transmission between cells, regulating immune responses, cell proliferation, differentiation, and apoptosis. Serum cytokine analysis in mice showed that the concentrations of interleukin 6 (IL-6), interleukin 10 (IL-10), and TGF-β in the S1, S5, and S6-treated groups were lower than those in the control group (Figure 7A–C). This indicated that therapeutic interventions could be effective in modulating cytokine levels within the tumor microenvironment, providing important insights and directions for cancer treatment research. Furthermore, tumor-associated macrophages (TAMs), which are present in the tumor microenvironment, significantly influence tumor development and treatment via their interactions with tumor cells. The proportion of TAMs in the tumor tissues of the S1-treated groups was significantly increased compared with that in the control group (Figure 7D and Figure S3).

## 3. Discussion

Immune checkpoint inhibitors, particularly anti-PD-1/PD-L1 antibodies, have transformed cancer treatment by enabling clinical remission in advanced tumors such as non-small cell lung cancer, melanoma, and ovarian cancer [19,20,21,22,23,24,25]. However, primary and acquired resistance are still unresolved obstacles [3,26,27]. The expression levels of Siglec-15 and PD-L1 are mutually exclusive [10]. Its unique ability to modulate the immune response suggests that Siglec-15 might be a therapeutic target with reduced side effects [28].

The generation of chimeric heavy-chain antibodies by fusing camelid nanobodies with human IgG-Fc highlights the advantages of this platform. The small size of nanobodies likely enhances tissue penetration, while Fc fusion confers effector functions such as antibody-dependent cellular cytotoxicity (ADCC) and prolonged serum half-life. Our study showed that S1, S5, and S6 exhibited good blocking activities in vitro, demonstrating potential for therapeutic applications. S1 demonstrated exceptional binding affinity to human and murine Siglec-15 (EC_50_ of 0.04 nM), which was nearly 2-fold lower than that of a previously reported high-affinity anti-Siglec-15 antibody (EC_50_ of 0.07665 nM) [29]. Notably, the ability of S1, S5, and S6 for Siglec-15 to disrupt interactions with key ligands (CD44, MAG, sialyl-Tn, and LRR4C) suggests a dual mechanism of action: direct blockade of Siglec-15-mediated immunosuppressive signaling and potential modulation of ligand-dependent tumor-stroma crosstalk. The superior potency of S1 may arise from its unique binding epitope or higher affinity, warranting further structural characterization.

In vivo, S1-driven tumor suppression correlated with significant remodeling of the tumor microenvironment, marked by reduced levels of immunosuppressive cytokines (TGF-β, IL-6, IL-10) and increased infiltration of TAMs. While elevated TAMs are often associated with pro-tumorigenic phenotypes, the observed anti-tumor effects suggest S1 may promote their polarization toward an M1-like, immunostimulatory state. This hypothesis is supported by the cytokine profile shifts, as TGF-β and IL-10 are known to drive M2 macrophage differentiation. Further single-cell analyses are needed to delineate macrophage subsets and functional states post-treatment.

A monoclonal antibody targeting Siglec-15 (NC318) has shown promising results in a variety tumor model in mice [10], and there is an ongoing phase I/II clinical trial (NCT03665285) targeting advanced or metastatic solid tumors, which has shown responses in patients suffering from non-small-cell lung cancer who were unresponsive to PD-1 inhibitors. However, the clinical trial of NC318 monotherapy for non-small cell lung cancer was terminated, possibly because of irrational enrollment criteria. Despite promising data from in vitro and mouse model studies, the clinical trials have not met expectations, suggesting the need to adjust the inclusion criteria or explore combination therapies with anti-PD-1 antibodies. Therefore, a phase II clinical trial of NC318 in combination with Keytruda (a humanized antibody recognizing PD-1) for non-small cell lung cancer was conducted [30]. The anti-Siglec-15 antibody developed in this study has potential for engineering modifications. Subsequent studies could focus on genetically engineering this anti-Siglec-15 antibody alongside anti-PD-1 antibodies, representing a new strategy for cancer immunotherapy.

While our data suggest a link between Siglec-15 blockade and cytokine reduction, there are several limitations that require further consideration. First, the study utilized murine tumor models, and the translatability of these findings to human cancers remains to be validated. Second, the in vivo evaluation was performed in immunodeficient BALB/c nude mice, which precluded analysis of T-cell mediated immune responses. Third, long-term safety evaluations, particularly regarding autoimmune sequelae from sustained Siglec-15 blockade, are critical given its low but detectable expression in normal tissues like osteoclasts.

In conclusion, the anti-Siglec-15 chimeric heavy-chain antibodies was identified and evaluated. The marked anti-tumor activity of S1, mediated through tumor microenvironment reprogramming and immune activation, justifies its progression toward clinical development. Future work should prioritize mechanistic studies of macrophage polarization and combinatorial therapeutic strategies to maximize patient benefit.

## 4. Materials and Methods

### 4.1. Animals and Cell Lines

An adult male healthy Bactrian camel was purchased from Deschamps Camel Breeding Co. (Hohhot, Inner Mongolia, China). Female-specific pathogen-free (SPF), six-week-old female BALB/c nude mice were obtained from Beijing Spefo Biotechnology Co. (Beijing, China) and housed under standard conditions (22 ± 1 °C, 55 ± 5% humidity, 12 h light/dark cycle) with free access to food and water.

FreeStyle^TM^ HEK293-F cells, NCI-H157 cells, 293T cells were purchased from Thermo Fisher Scientific (Waltham, MA, USA). NCI-H157-S15 cells (human non-small cell lung adenocarcinoma cells with high expression of Siglec-15) was kindly gifted by Prof. Jiannan Feng from the Institute of Pharmacology and Toxicology (Beijing, China). All cell culture reagents were sourced from Thermo Fisher Scientific (Waltham, MA, USA). FreeStyle^TM^ HEK293-F cells were cultured with FreeStyle™ 293 medium (Thermo Fisher Scientific, Waltham, MA, USA) at 37 °C with 5% CO_2_ and passaged every 2–3 days. NCI-H157 and 293T cells were maintained in RPMI 1640 (Thermo Fisher Scientific, Waltham, MA, USA) or DMEM (Thermo Fisher Scientific, Waltham, MA, USA), respectively, each supplemented with 10% FBS. NCI-H157-S15 cells used RPMI 1640 medium with 10% FBS and 1 μg/mL puromycin. All cell lines were incubated at 37 °C with 5% CO_2_ and passaged at 80–90% confluence using 0.25% trypsin.

### 4.2. Immunization of the Camel

The camel was immunized with recombinant human Siglec-15 protein (SinoBiological, Beijing, China) in Freund’s complete adjuvant (Sigma-Aldrich, Louis, MO, USA) for the initial subcutaneous multi-site injections. Subsequent immunizations used Freund’s incomplete adjuvant (Sigma-Aldrich, Louis, MO, USA) every 14 days. After the fourth immunization, camel serum was collected using enzyme linked immunosorbent assay (ELISA) to evaluate the specific antibody titer, using pre-immunization serum as the blank control. Booster immunization was administered when the desired antibody titer was reached [31].

### 4.3. Construction of the Phage Display Nanobody Library

Peripheral blood was collected from camels after the fifth immunization and peripheral blood lymphocytes (PBMC) were isolated using a lymphocyte isolation solution. Total RNA from PBMC was extracted using Total RNA kit I (Omega Bio—tek, Norcross, GA, USA) and then reverse transcribed to synthesise cDNA. Amplification of nanobody gene fragments was achieved by two rounds of PCR. Nanobody gene fragments were ligated into NEN-SCFV vector using conventional molecular cloning techniques. The ligation product was electro-transformed into *Escherichia coli* TG1 cells to establish an anti-Siglec-15 phage display nanobody library. Then they were spread on 2-YT-GA plates (with 100 μg/mL ampicillin and 2% glucose) and a number of single colonies were selected for sequencing analysis, which was used to assess correct insertion rates and library diversity.

### 4.4. Panning and Screening of the Phage Display Nanobody Library

Then, 200 ng/well of recombinant Human Siglec-15 protein was coated into the immunotube with carbonate buffer (pH = 9.6) and incubated at 4 °C overnight. The amount of antigen coated was reduced by half with each increase in the number of screening rounds. After washing five times with PBST (phosphate buffered saline (PBS) containing 1% Tween-20), PBS containing 2% bovine serum albumin (BSA) was added and incubated for 2 h at 37 °C. After washing ten times with PBST, the nanobody phage display library was added and incubated for 2 h at room temperature. Elution was performed by adding 0.1 M HCl-Glycin (pH = 2.2) followed by neutralization with 1 M Tris-HCl. To the neutralized eluate was added the TG1 bacterial solution cultured on the same day (OD_600_ ≈ 0.6) and incubated for 30 min at 37 °C, 150 r/min. The bacterial solution was spread on 2-YT-GA plates and incubated inverted overnight at 37 °C and then used to calculate the titer of the phage antibody library. The recombinant human Siglec-15 protein was coated into 96-well ELISA microtiter plates (Costar, Corning, NY, USA) with carbonate buffer, and adjacent columns of each antigen group were coated with irrelevant antigens as a negative control at 4 °C overnight. After washing six times with PBST, PBS containing 2% BSA was added and incubated at 37 °C for 2 h. Single phage colonies were added into the plate and incubated at 37 °C for 1.5 h. After washing six times with PBST, HRP-labeled M13 mouse monoclonal antibody (1:4000, SinoBiological, Beijing, China) was added and incubated at 37 °C for 1 h. Following another six washes with PBST, plates were added with o-phenylenediamine chromogen substrate (Sigma-Aldrich, Louis, MO, USA) and incubated in the dark for 10 min. The reaction was stopped by adding 2 M H_2_SO_4_ and the absorbance was measured at 492 nm and 630 nm using a microplate reader (Molecular Devices, Sunnyvale, CA, USA). The clones whose absorbance in the experimental group was three times or more that in the control group were identified as positive clones. The positive clones were sequenced and subjected to further analysis.

### 4.5. Construction, Expression and Purification of the Chimeric Heavy-Chain Antibodies

The positive clones screened from the nanobody phage display library were used as templates, and primers were designed to amplify the VHH gene fragment, which was inserted into the eukaryotic expression vector pTSE-hFc containing human IgG-Fc (hFc) to construct the VHH-hFc fusion protein expression plasmid. Plasmids were transfected into FreeStyle^TM^ HEK293-F cells for transient expression by FectoPRO transfection reagent. After purification using a HiTrap MabSelect Xtra column (Cytiva, Marlborough, MA, USA), sodium dodecyl sulfate-polyacrylamide gel electrophoresis (SDS-PAGE) was employed to determine the antibodies’ molecular weight and purity.

### 4.6. Binding Activity and Specificity Assays

For the binding activity assay, 200 ng/well of recombinant human and murine Siglec-15 proteins were coated onto ELISA plates and incubated at 4 °C overnight. After washing six times with PBST, PBS containing 3% skimmed milk powder was added and incubated at 37 °C for 1.5 h. Discarded the liquid in the plate, added the antibody solution (starting concentration 80 μg/mL) diluted according to a 3-fold multiplicity ratio, and set up a total of 15 gradients to incubated for 1.5 h at 37 °C. Horseradish peroxidase (HRP)-labeled goat anti-human IgG (1:4000, Abcam, Cambridge, UK) was added and incubated at 37 °C for 1 h. Following another six washes with PBST, plates were added with o-phenylenediamine chromogen substrate and incubated in the dark for 10 min. The reaction was stopped by adding 2 M H_2_SO_4_ and the absorbance was measured at 492 nm and 630 nm using a microplate reader.

The ELISA for the antibody specificity assay was performed as described above except that the antibodies (15 μg/mL) were added at 100 μL/well.

For cell-based ELISA, NCI-H157-S15 cells were inoculated at 2 × 10^4^ cells/well in 96-well plates and incubated overnight. The remaining steps followed the ELISA procedure described above except that the temperature was 4 °C.

Antibody affinity was determined using the ForteBIO^®^ Octet Qk^e^ System (Pall ForteBio, Fremont, CA, USA), based on biolayer interferometry (BLI). We then added 200 μL of HBS-EP^+^ buffer (Cytiva, Marlborough, MA, USA) to each well in the probe fixation cassette, and soaked the AHC (Anti-human IgG Fc) probe in the fixation cassette for 30 min. HBS-EP buffer was used to dilute the antibodies to 200 nM, followed by fixation on Anti-hIgG Fc Capture biosensors. The biosensors were immersed in HBS-EP for baseline determination, followed by incubation in diluted recombinant Human Siglec-15 protein (diluted to 500 nM, 250 nM, 125 nM, 62.5 nM, 31.25 nM, 15.6 nM) for association, and lastly by a dissociation step. Data Analysis 7.0 software (Pall ForteBio, Fremont, CA, USA) was used to analyze the data and plot the kinetic curve [32].

### 4.7. Competitive Binding Assay

The ForteBIO^®^ Octet Qk^e^ System (Pall ForteBio, Fremont, CA, USA) was utilized to carry out the competitive binding assays. Antibodies (400 nM in HBS-EP buffer) were loaded onto Anti-hIgG Fc Capture biosensors, rinsed for 1 min in HBS-EP buffer, added to the sample plate, and incubated for 10 min with 400 nM Siglec-15 or HBS-EP buffer for association. A biosensor loaded with control human IgG was used to determine the background binding between Siglec-15 and the sensor for quality control. Data Analysis Software 7.0 was used to analyze the data [32].

### 4.8. Blocking Activity Assay

Then, 200 ng/well of recombinant human cluster of differentiation 44 (CD44, Novoprotein, Suzhou, China) or myelin-associated glycoprotein (MAG, Novoprotein, Suzhou, China) were coated onto 96-well plates. Then, 50 μL/well of biotin-labeled Siglec-15-Fc (100 μg/mL, Novoprotein, Suzhou, China) and 50 μL/well of antibodies (100 μg/mL) were added to each well. Then, HRP-labeled streptavidin (1:6000, Thermo Fisher Scientific, Waltham, MA, USA) was added to detect the biotin-labeled Siglec-15-Fc. The remaining steps followed the ELISA procedure described above. The formula used was: Blocking rate = [1 − (antibody group − blank group)/(positive group − blank group)] × 100.

NCI-H157 cells in logarithmic growth phase were digested with 0.25% trypsin, centrifuged at 300× *g*, and the cell concentration was adjusted to 5 × 10^5^/mL with PBS. One tube of cells was spiked with Anti-sialyl Tn antibody (Abcam, Cambridge, UK) and DyLight^®^ 488 goat anti-mouse IgG (H + L) antibody (Abcam, Cambridge, UK), and one tube of cells was spiked with DyLight^®^ 488 goat anti-mouse IgG (H + L) antibody. Siglec-15-Biotin (100 μg/mL, Novoprotein, Suzhou, China) and 50 μL of each of the three antibodies (100 μg/mL) were added to the cells, and the cells were incubated for 30 min at 4 °C. Subsequently, Allophycocyanin (APC)-labeled Streptavidin (1:3000, Biolegend, San Diego, CA, USA) was added to wells, followed by analysis using flow cytometry.

The LRRC4C expression plasmid was transfected into 293T cells in logarithmic growth phase by Lipofectamine 2000 (Thermo Fisher Scientific, Waltham, MA, USA) transfection reagent and cultured for 72 h. Cells were digested with 0.25% trypsin, and then the cell concentration was adjusted to 5 × 10^5^/mL with PBS. Siglec-15-Biotin (5 μg/mL) and 50 μL of each of the three antibodies (100 μg/mL) were added to the cells, and the cells were incubated for 30 min at 4 °C. Subsequently, Allophycocyanin (APC)-labeled Streptavidin (1:3000) was added to wells, followed by analysis using flow cytometry.

### 4.9. T-Cell Response Assays

Anti-human CD3 antibody (BioLegend, San Diego, CA, USA) was coated into 96-well plate with PBS and incubated at 4 °C overnight. PBMC from the blood of healthy donors were isolated using lymphocyte isolation solution, and PMBC (3 × 10^5^ cells/well) were inoculated into 96-well plates. Siglec-15-Biotin (5 μg/mL) and 50 μL of each of the three antibodies (50 μg/mL) were added to the 96-well plates and the 96-well plates were incubated for 72 h at 37 °C. The cell supernatant was harvested, and ELISA MAX Standard Set Human kit (IFN-γ, Biolegend, San Diego, CA, USA) was used to measure the levels of IFN-γ. Cell Titer-Glo (Promega, Madison, WI, USA) was added to the 96-well plate and cell viability was assayed.

### 4.10. Evaluation of Anti-Tumor Activity In Vivo

Six-week-old female BALB/c nude mice were subcutaneously inoculated with NCI-H157-S15 cells (3 × 10^6^ cells/mouse). After seven days, mice with tumors were randomly assigned to groups of six. PBS served as the control group, while S1, S5, and S6 (10 mg/kg each) were the treated groups. The antibodies were administered once every two days via tail vein administration, with ongoing monitoring of tumor size and mouse body weight. The tumor volume was calculated as follows: volume = length × (width^2^)/2.

When the tumor volume of mice was approximately 100 mm^3^, the mice were randomly divided into three groups. S1, S5, and S6 (labelled with Alexa Fluor 750 dye, Thermo Fisher Scientific, Waltham, MA, USA) were injected through the tail vein. Images were taken at 1 h, 12 h, 24 h, and 72 h after injection using a small animal live imaging system.

After treatment completion, blood samples were collected from the mice via the retro-orbital sinus to obtain serum. Serum cytokine levels were measured using a transforming growth factor beta (TGF-β) ELISA assay kit (Biolegend, San Diego, CA, USA) and a Mouse Luminex^®^ Discovery Assay (R&D Systems, Minneapolis, MN, USA).

Mice were euthanized post-treatment, and tumor tissues were dissected, weighed, and imaged before being placed in Dulbecco’s modified Eagle’s medium (DMEM, Gibco, Grand Island, NY, USA). Digestion was carried out using a solution of DMEM with 1 mg/mL collagenase I and 200 μg/mL DNase I (Sigma-Aldrich, Louis, MO, USA) at 37 °C for 30 min. After centrifugation and adjustment of the cell density to 2 × 10^7^ cells/mL, the cells were added to the wells of a 96-well plate and incubated with anti-Fc receptor antibodies(3:100, Biolegend, San Diego, CA, USA) at 4 °C. Anti-mouse-CD45.2 antibodies, anti-mouse-Ly6C antibodies, anti-mouse Ly-6G antibodies, anti-mouse-CD11b antibodies, and anti-mouse F4/80 antibodies (final concentration 4 μg/mL, all from Biolegend, San Diego, CA, USA) were added to the plates, incubated at 4 °C for 30 min, and analyzed using flow cytometry [33,34].

### 4.11. Statistical Considerations

Analyses were conducted with R (4.3.1). Graphs were created using GraphPad Prism 8 software (GraphPad inc., La Jolla, CA, USA). Quantitative data are presented as mean ± standard deviation (SD). Normality was assessed using the Shapiro–Wilk test, and homogeneity of variances was verified via Levene’s test. For data meeting parametric assumptions, one-way ANOVA followed by Tukey’s multiple comparisons test was performed. For non-normally distributed data, the Kruskal–Wallis test was used, followed by Dunn’s multiple comparisons test. A *p*-value < 0.05 was considered statistically significant, with specific significance levels denoted as follows: * *p* < 0.05, ** *p* < 0.01, *** *p* < 0.001.

## Figures and Tables

**Table 1:** Enrichment analysis of the anti-Siglec-15 phage display nanobody library by three rounds of panning.

Round of Panning	Input (pfu)	Output (pfu)	Output/Input
Round 1	5.00 × 10^11^	2.80 × 10^5^	5.60 × 10^−5^
Round 2	3.00 × 10^11^	2.70 × 10^6^	9.00 × 10^−4^
Round 3	1.00 × 10^11^	1.60 × 10^8^	1.60 × 10^−3^

Input: the amount of input phage; Output: the amount of output phage. Output/Input, the amount of output phage/the amount of input phage.

**Table 2:** Dissociation constants of the antibodies.

Antibody	Mean			
	Kon (10^4^ Ms^−1^)	Kdis (10^−4^ s^−1^)	KD (10^−9^ M)	R^2^
S1	4.20	5.33	0.13	0.99
S5	5.73	8.92	0.16	0.99
S6	6.89	14.30	0.21	0.99

## Data Availability

The data of this study are available from the corresponding authors upon reasonable request.

## Supplementary Materials

The following supporting information can be downloaded at: https://www.mdpi.com/article/10.3390/ijms26115068/s1.
