# Supplementary material for: Anti-Tumor Activities of Anti-Siglec-15 Chimeric Heavy-Chain Antibodies
Source: Int J Mol Sci. 2025 May 24;26(11):5068. doi: 10.3390/ijms26115068 (PMC12154215; doi:10.3390/ijms26115068)
Supplement: Supplementary file 1 [file ijms-26-05068-s001.zip › ijms-3595993-supplementary.pdf]

*Article*

# Anti-Tumor Activities of Anti-Siglec-15 Chimeric Heavy-Chain Antibodies

**Kexuan Cheng [1,2,†], Jiazheng Guo [1,†], Yating Li [1,2], Qinglin Kang [1], Rong Wang [1], Longlong Luo [3], Wei Wang [2,*] and Jiansheng Lu [1,*]**

[1] Laboratory of Advanced Biotechnology, Beijing Institute of Biotechnology, Beijing 100081, China; 16696653458@163.com (K.C.); sdqzgjz@163.com (J.G.); lyt18214729367@163.com (Y.L.); kql_lynn@163.com (Q.K.); wangrong_8312@163.com (R.W.)

[2] College of Public Health, Zhengzhou University, Zhengzhou 450001, China

[3] State Key Laboratory of Toxicology and Medical Countermeasures, Institute of Pharmacology and Toxicology, 27 Taiping Road, Beijing 100850, China; luolong_long@126.com

* Correspondence: ww375@zzu.edu.cn (W.W.); yangzhixin@bmi.ac.cn (J.L.)

† These authors contributed equally to this work.

## Figure supplement legends:

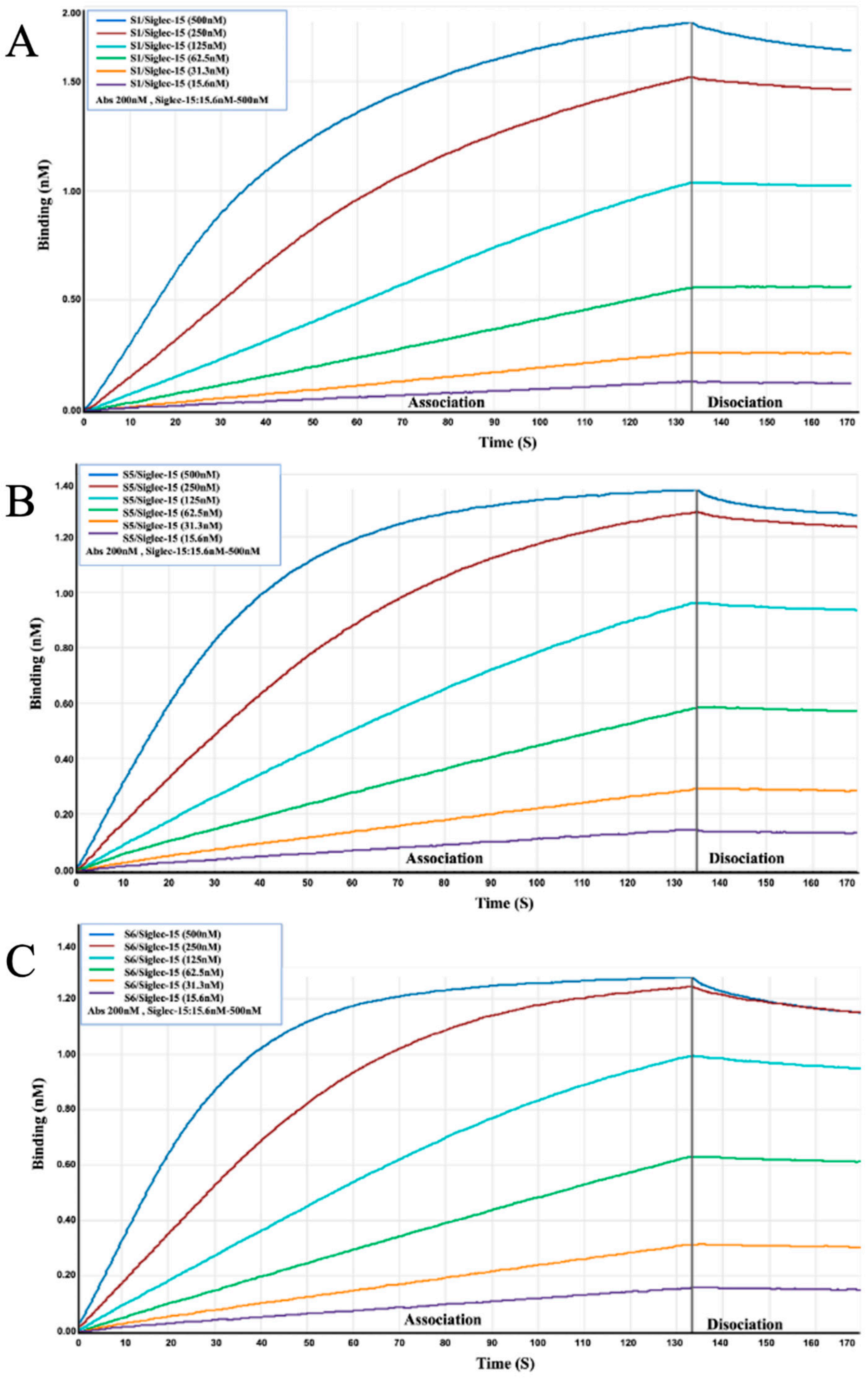


Figure S1 Detection of the kinetic processes of S1, S5, S6 and recombinant human Siglec-15 protein. The kinetics processes of S1 (A), S5 (B), and S6 (C) binding to Siglec-15.

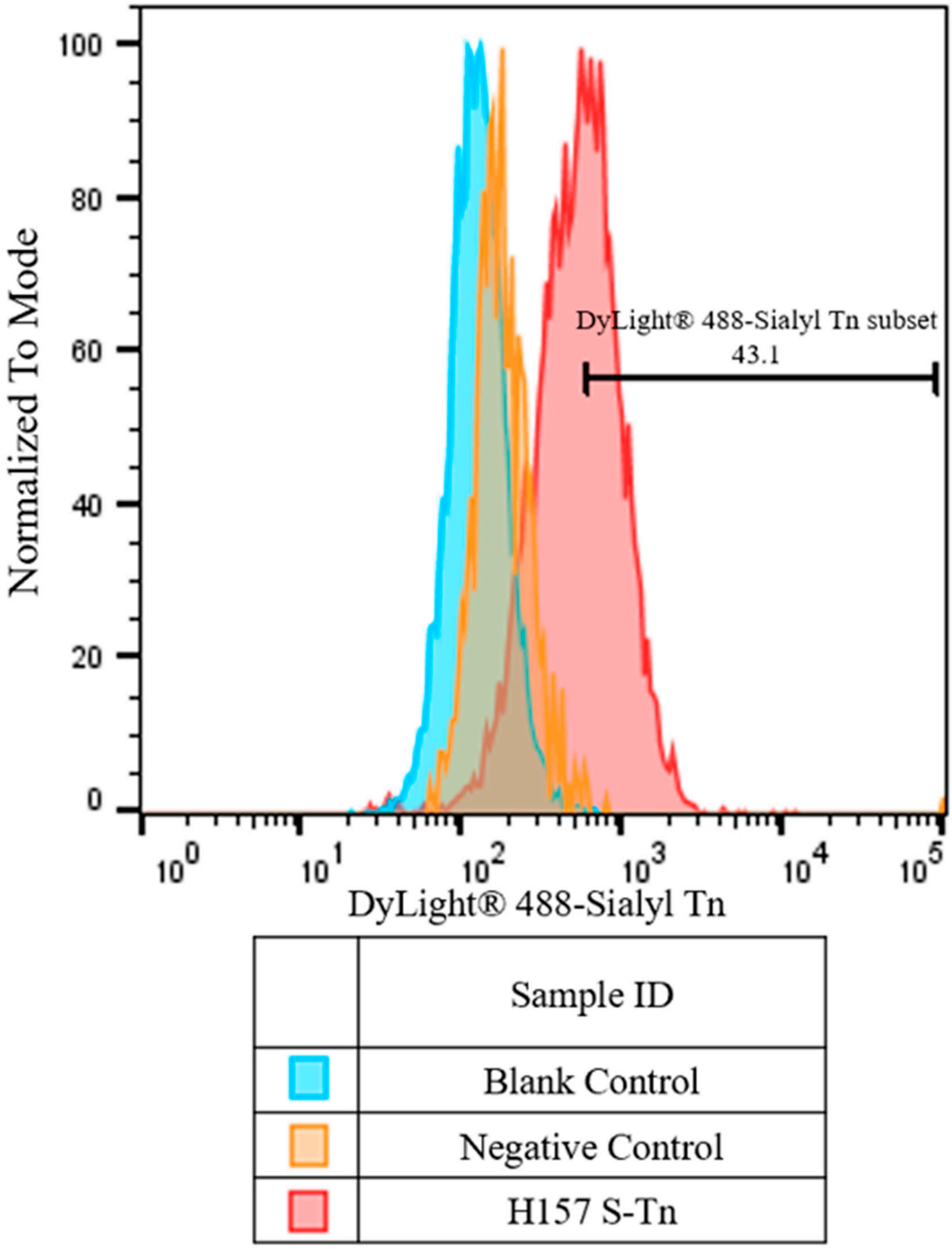


Figure S2 The expression of sialyl-Tn on the surface of NCI-H157 cells was detected using flow cytometry.

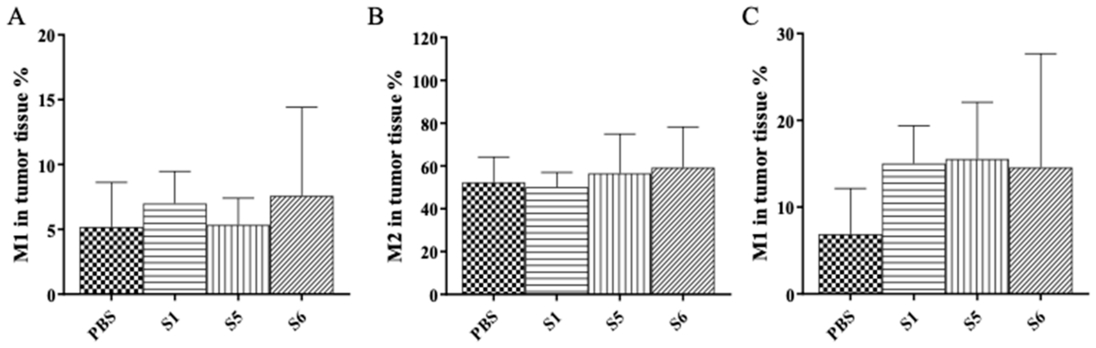


Figure S3 A. CD80+/CD86+ M1 macrophage percentage. B. CD206+/CD163+ M2 macrophage percentage. C. MHC-II IA/IE+ M1 macrophage percentage.
